# Dose exploration results from Phase 1 study of cemiplimab, a human monoclonal programmed death (PD)-1 antibody, in Japanese patients with advanced malignancies

**DOI:** 10.1007/s00280-020-04161-6

**Published:** 2020-11-04

**Authors:** Shigehisa Kitano, Toshio Shimizu, Takafumi Koyama, Takahiro Ebata, Satoru Iwasa, Shunsuke Kondo, Akihiko Shimomura, Yutaka Fujiwara, Noboru Yamamoto, Anne Paccaly, Siyu Li, Petra Rietschel, Tasha Sims

**Affiliations:** 1 Department of Experimental Therapeutics Koto-ku, National Cancer Center Hospital, 5-1-1, Tsukiji, Chuo-ku, Tokyo, 104-0045 Japan; 2 Regeneron Pharmaceuticals, Inc., Tarrytown, NY USA; 3 Regeneron Pharmaceuticals, Inc., Basking Ridge, NJ USA

**Keywords:** Cemiplimab, Anti–PD-1, Immunotherapy, Advanced tumors, Japanese patients

## Abstract

**Purpose:**

Part 1 of this two-part, open-label, Phase 1 study (NCT03233139) assessed the safety, tolerability, pharmacokinetics, immunogenicity, and clinical activity of cemiplimab in Japanese patients with advanced malignancies.

**Methods:**

Patients received cemiplimab 250 mg (*n* = 6) or 350 mg (*n* = 7) every 3 weeks intravenously for up to 108 weeks in Part 1. Tumor responses were assessed by investigators every 9 weeks using the Response Evaluation Criteria in Solid Tumors version 1.1.

**Results:**

Of 13 patients enrolled, median age was 62 years (range 33–75) and eight patients were female. Median duration of cemiplimab exposure was 13.1 weeks (range 3.0‒113.6). At the time of data cut-off, 11 patients (84.6%) had discontinued treatment (majority due to disease progression: *n* = 8, 61.5%). The most common treatment-emergent adverse events (TEAEs) of any grade were contact dermatitis, rash, and viral upper respiratory tract infection (each *n* = 3, 23.1%). Five grade ≥ 3 TEAEs were reported in four patients: autoimmune colitis, dehydration, hyponatremia, hypophosphatemia, and muscular weakness. No dose-limiting toxicities were reported and no TEAEs led to death. Cemiplimab concentrations in serum were consistent with previously reported pharmacokinetic characteristics of cemiplimab. No anti-drug antibodies were detected in serum. Objective response rate [ORR; complete response + partial response (PR)] was 30.8% (four PR) and disease control rate [ORR + stable disease (SD)] was 46.2% (6/13; two SD).

**Conclusion:**

Cemiplimab exhibited antitumor activity in Japanese patients with advanced malignancies. The safety profile was comparable to those previously reported for cemiplimab and other PD-1 inhibitors.

**Trial registration:**

NCT03233139 at ClinicalTrials.gov.

**Graphic abstract:**

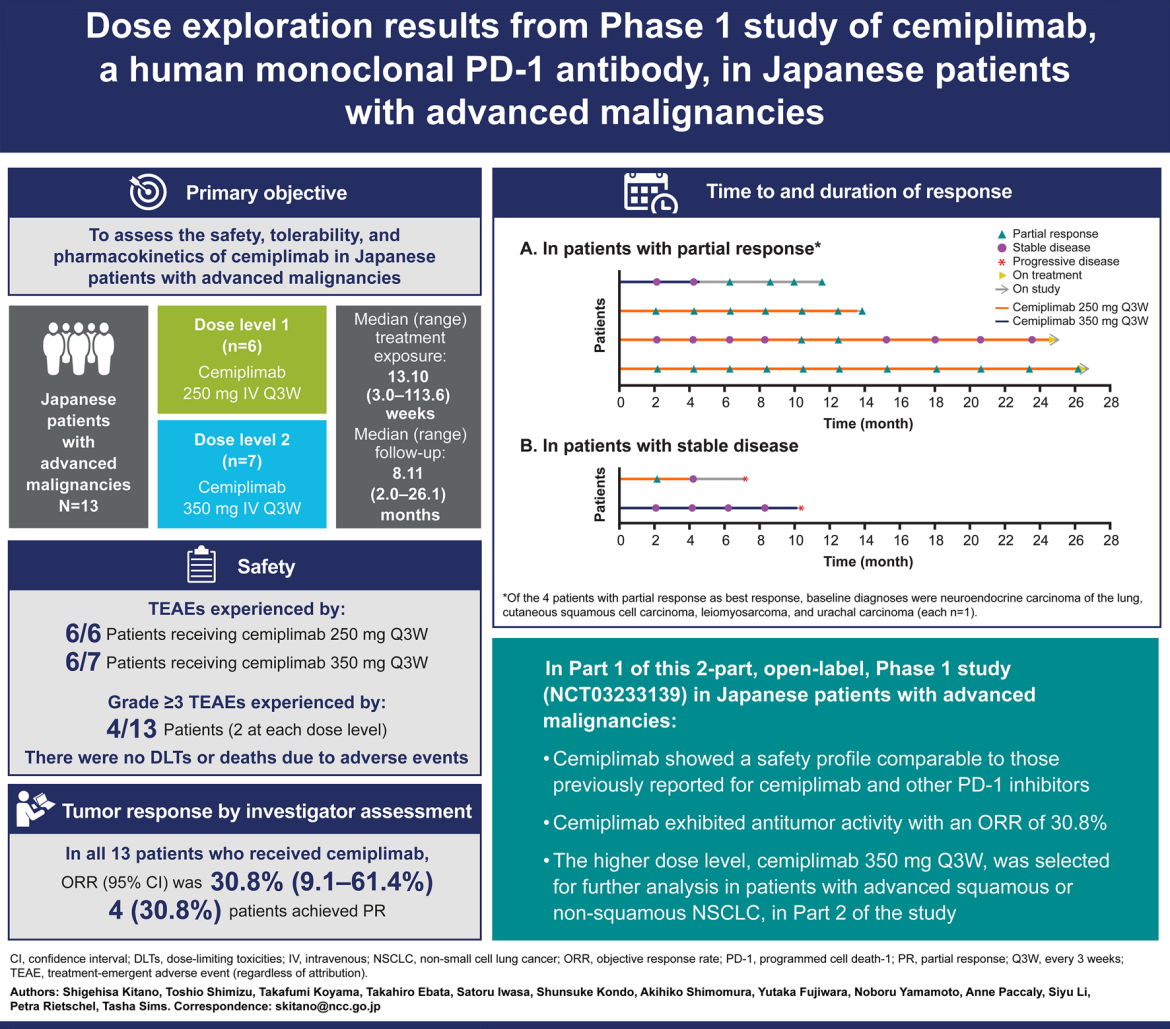

**Electronic supplementary material:**

The online version of this article (10.1007/s00280-020-04161-6) contains supplementary material, which is available to authorized users.

## Introduction

Cemiplimab is a high affinity, human, hinge-stabilized IgG4 monoclonal antibody to the programmed death (PD)-1 receptor that potently blocks the interactions of PD-1 with PD-ligand (L)1 and PD-L2 [1, 2]. It binds to PD-1 with high affinity and specificity. In preclinical studies, cemiplimab does not induce antibody-dependent cell-mediated cytotoxicity or complement-dependent cytotoxicity [1]. As a human antibody, it has a lower risk of inducing anti-drug antibodies (ADAs) than murine/human chimeric or humanized antibody therapies; and, thus, can potentially minimize immunogenicity risks compared with those antibody therapies [3]. Indeed, relatively low incidence of ADA was observed with cemiplimab (1.3%). This rate is comparable to, and in some cases numerically lower than, those observed with PD-1 or PD-L1 inhibitors (pembrolizumab: 0.7‒2.5%; durvalumab: 2.9‒6.6%; avelumab: 4.1‒5.9%; nivolumab: 4.1‒37.8%; atezolizumab: 30‒48%) after single or combination therapy [3].

In patients outside of Japan, cemiplimab has demonstrated a safety profile comparable to those for other PD-1 inhibitors and substantial anti-tumor activity in advanced malignancies, including cutaneous squamous cell carcinoma (CSCC) and non-small cell lung cancer (NSCLC), in controlled clinical trials and an observational study [2, 4, 5].

CSCC is the second most common skin cancer affecting Japanese and the worldwide population [6–9]. Until the emergence of PD-1 inhibitors, the prognosis was poor for patients with either locally advanced CSCC not amenable to surgery or metastatic CSCC [10–12]. Cemiplimab (“cemiplimab-rwlc” in the US) is the first therapy approved in the US, Europe, Canada, Australia, Brazil, Switzerland, and Israel for the treatment of patients with metastatic or locally advanced CSCC who are not candidates for curative surgery or curative radiation, with an objective response rate (ORR) of 47.2% [13, 14]. There is currently no approved therapy for CSCC in Japan. Most cases of CSCC in Japan are classified as high risk, warranting research of novel therapies [15].

High unmet needs for treatment of NSCLC exist both in Japan and worldwide. PD-1 inhibitors have quickly emerged as a treatment option with improved prognosis [16]. Interim data from a Phase 1 study of cemiplimab showed an acceptable safety profile and demonstrated antitumor activity in non-Japanese patients with NSCLC who had relapsed after or were refractory to first or further lines of therapy and for whom palliative radiotherapy was clinically indicated, which prompted pivotal trials of cemiplimab as monotherapy or in combination with other treatments in patients with stage IIIB, IIIC, or IV NSCLC [5].

This two-part Phase 1 study (NCT03233139) evaluates safety, tolerability, and pharmacokinetics (PK) of cemiplimab in Japanese patients with advanced malignancies. Tumor responses to cemiplimab treatment were also assessed. We report here dose exploration results from Part 1.

## Methods

### Study design

**Fig. 1:**
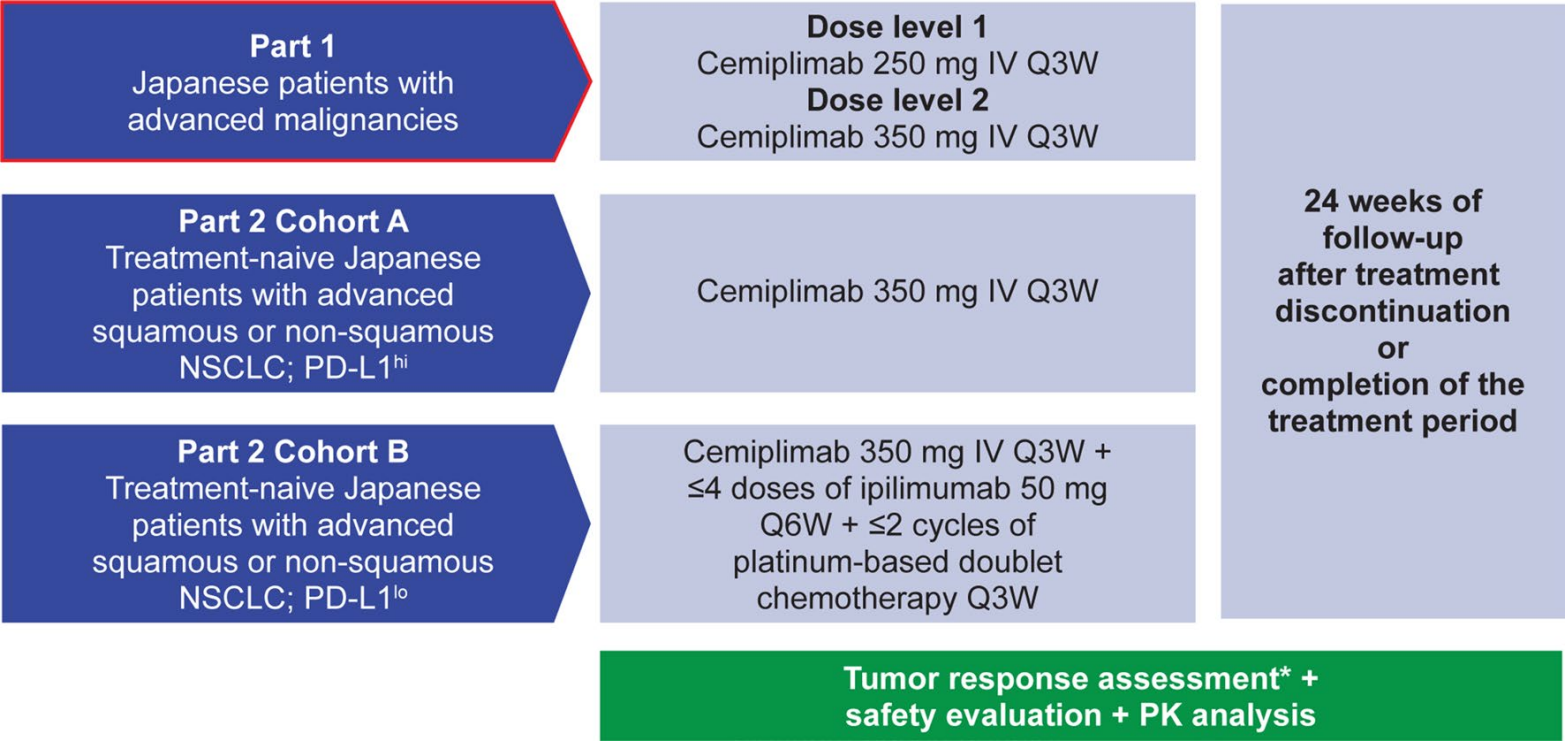
Study design. *Tumor responses were assessed using Response Evaluation Criteria in Solid Tumors version 1.1 by investigators in Part 1 and Part 2 Cohort B and by an independent central review committee in Part 2 Cohort A every 9 weeks in the first year, every 12 weeks in the second year, and every 8 weeks during the follow-up. *IV* intravenously, *NSCLC* non-small cell lung cancer, *PD-L1* programmed cell death-ligand 1, *PD-L1*^*hi*^ ≥ 50% PD-L1 expression in tumor cells, *PD-L1*^*lo*^ < 50% PD-L1 expression in tumor cells, *PK* pharmacokinetics, *Q3W* every 3 weeks, *Q6W* every 6 weeks

This two-part, open-label, Phase 1 study in Japanese patients comprises Part 1 of advanced malignancies treated with cemiplimab monotherapy [250 mg every 3 weeks (Q3W) or 350 mg Q3W] and Part 2 of advanced squamous or non-squamous NSCLC treated with cemiplimab (350 mg Q3W) alone or with standard of care platinum-based doublet chemotherapy for 2 cycles and ipilimumab 50 mg every 6 weeks (Q6W) for up to four doses (Fig. 1). Part 1 was conducted at National Cancer Center Hospital, Tokyo, Japan. Part 2 is being conducted at multiple centers in Japan and consists of cohorts A (PD-L1 expression in tumor cells ≥ 50%) and B (PD-L1 expression in tumor cells < 50%). The data cut-off date of the dose exploration results in Part 1 reported here was September 6, 2019. Part 2 of the study is ongoing.

### Patients, treatment, and follow-up

Adult patients 20 years of age or older were eligible for Part 1 enrollment. Key inclusion criteria for Part 1 were histologically or cytologically confirmed diagnosis of malignancy with no alternative standard of care therapeutic option; Eastern Cooperative Oncology Group (ECOG) performance status of 0 or 1; adequate hepatic, renal, and bone marrow functions. Adequate hepatic function was defined as total bilirubin ≤ 1.5 × upper limit of normal (ULN) or ≤ 3 × ULN if liver metastases; aspartate aminotransferase (AST) and alanine aminotransferase (ALT) ≤ 3 × ULN (or ≤ 5.0 × ULN if liver metastases or hepatocellular carcinoma). Patients with hepatic metastases or hepatic malignancies were eligible for enrollment, unless with concomitant AST ≥ 3 × ULN and/or ALT ≤ 5 × ULN, and total bilirubin of 1.5‒3 × ULN. Adequate renal function was defined as serum creatinine ≤ 1.5 × ULN or creatinine clearance > 50 mL/min (or estimated glomerular filtration rate > 30 mL/min × 1.73 m^2^ if renal cell carcinoma). Adequate bone marrow function was defined as hemoglobin ≥ 8.0 g/dL; absolute neutrophil count ≥ 1.5 × 10^9^/L; platelet count ≥ 75 × 10^9^/L. In addition, patients must have been born in Japan, and their biological parents and grandparents must be of Japanese origin.

Patients were excluded from Part 1 of the study if they received prior treatment targeting the PD-1/PD-L1 pathway. Additional key exclusion criteria included, but were not limited to: ongoing or recent autoimmune disease that required systemic immunosuppressive treatments; treatment with corticosteroids (> 10 mg prednisone daily or equivalent) within the first 4 weeks prior to the first dose of cemiplimab; active brain metastases; and active uncontrolled human immunodeficiency virus, hepatitis C virus, or hepatitis B virus infections.

All patients in Part 1 received cemiplimab 250 mg or 350 mg Q3W as a 30-min intravenous infusion on Day 1 of each treatment cycle for up to 2 years of treatment, or until completion of treatment or progression of disease, unacceptable toxicity, withdrawal of consent, or meeting of another study withdrawal criterion. Patients had a follow-up for up to 24 weeks after the treatment period.

### Objectives

The primary objective of the study was to assess the safety, tolerability, and PK of cemiplimab in Japanese patients with advanced malignancies. The secondary objective of the study was to assess the immunogenicity of cemiplimab. The exploratory objective of Part 1 was to evaluate tumor response to cemiplimab monotherapy in patients with measurable disease.

### Assessments

Severity of adverse events (AEs) was graded according to the National Cancer Institute Common Terminology Criteria for Adverse Events (version 4.03) [17]. The relatedness of AEs to treatment was assessed by investigators. PK of cemiplimab was assessed after the first dose. Trough and end-of-infusion concentrations of cemiplimab in serum were measured upon multiple dosing throughout the study using a validated enzyme-linked immunosorbent assay with a lower limit of quantification of 0.078 mg/L. ADAs against cemiplimab in serum were measured at pre-dose and during treatment using a validated electrochemiluminescence bridging immunoassay. Tumor responses were assessed using Response Evaluation Criteria in Solid Tumors version 1.1 (RECIST 1.1) [18] by investigators in Part 1 every 9 weeks in the first year, every 12 weeks in the second year, and every 8 weeks during the 24-week follow-up period.

### Statistical analysis

No statistical hypothesis was tested in this observational study. For Part 1, the sample size of approximately 14 patients (up to seven patients per dose group) was selected based on modified 3 + 3 design (4 + 3). The safety and efficacy analysis sets included all patients who received at least one dose of cemiplimab.

## Results

### Patients, treatment, and follow-up

Of the 13 patients with advanced malignancies enrolled in Part 1, the median age was 62.0 years (range 33‒75), eight patients (61.5%) were female, the majority (8/13; 61.5%) had ECOG performance status of 0, 12 (92.3%) had prior cancer-related systemic therapy, seven (53.8%) had prior cancer-related radiation, and nine (69.2%) had prior cancer-related surgery (Table 1). Patients who received 350 mg Q3W were slightly older and had higher ECOG performance status versus those who received 250 mg Q3W. At the time of data cut-off, 11 patients (84.6%) discontinued treatment and two (15.4%) remained on treatment. No patients completed treatment. The most common reason for treatment discontinuation was disease progression (8/13, 61.5%). Median number of administered doses of cemiplimab was 4.0 (range 1–36) and median duration of exposure was 13.10 weeks (range 3.0–113.6) (Supplementary Table 1). Median duration of follow-up at the time of data cut-off was 8.11 months (range 2.0–26.1).

**Table 1:** Patient demographics and baseline characteristics

	Cemiplimab 250 mg Q3W (*n* = 6)	Cemiplimab 350 mg Q3W (*n* = 7)	Total (*N* = 13)
Median age, years (range)	55.5 (33‒75)	64.0 (45‒74)	62.0 (33‒75)
≥ 65 years, *n* (%)	2 (33.3)	3 (42.9)	5 (38.5)
Female, *n* (%)	3 (50.0)	5 (71.4)	8 (61.5)
ECOG performance status, *n* (%)			
0	5 (83.3)	3 (42.9)	8 (61.5)
1	1 (16.7)	4 (57.1)	5 (38.5)
Primary tumor site, *n* (%)			
Lung	1 (16.7)	1 (14.3)	2 (15.4)
Bladder	1 (16.7)	0	1 (7.7)
Breast	0	1 (14.3)	1 (7.7)
Non-melanoma skin	1 (16.7)	0	1 (7.7)
Urethra	0	1 (14.3)	1 (7.7)
Uterus	1 (16.7)	0	1 (7.7)
Ovary	1 (16.7)	0	1 (7.7)
Prostate	0	1 (14.3)	1 (7.7)
Pancreas	0	1 (14.3)	1 (7.7)
Other	1 (16.7)	2 (28.6)	3 (23.1)
Prior cancer-related radiation, *n* (%)	3 (50.0)	4 (57.1)	7 (53.8)
Median number of prior cancer-related radiation (range)	0.5 (0‒1)	1.0 (0‒2)	1.0 (0‒2)
Prior cancer-related systemic therapy, *n* (%)	5 (83.3)	7 (100)	12 (92.3)
Median number of prior cancer-related systemic therapy (range)	3.0 (0‒15)	3.0 (1‒9)	3.0 (0‒15)
Prior cancer-related surgery, *n* (%)	4 (66.7)	5 (71.4)	9 (69.2)
Median number of prior cancer-related surgeries (range)	1.5 (0‒5)	1.0 (0‒5)	1.0 (0‒5)

*ECOG* Eastern Cooperative Oncology Group, *Q3W* every 3 weeks

### Safety

Twelve patients (92.3%) experienced at least one treatment-emergent AE (TEAE) of any grade, regardless of attribution of relatedness to study drug, during the treatment period (Table 2). TEAEs occurred in six patients (100.0%) treated with cemiplimab 250 mg Q3W and six (85.7%) treated with 350 mg Q3W. The most common TEAEs were contact dermatitis, rash, and viral upper respiratory tract infection (each 3/13; 23.1%). Four patients (30.8%; two at each dose level) experienced a total of five grade ≥ 3 TEAEs. Each of the following grade ≥ 3 TEAEs occurred once (with investigator assessment of treatment relatedness): autoimmune colitis (250 mg Q3W; related to treatment), dehydration (250 mg Q3W; related to treatment), hyponatremia (350 mg Q3W; unrelated to treatment), hypophosphatemia (250 mg Q3W; unrelated to treatment), and muscular weakness (350 mg Q3W; related to treatment).

**Table 2:** Treatment-emergent adverse events, regardless of attribution

*n* (%)	Cemiplimab 250 mg Q3W (*n* = 6)	Cemiplimab 350 mg Q3W (*n* = 7)	Total (*N* = 13)
Any	6 (100.0)	6 (85.7)	12 (92.3)
Grade ≥ 3	2 (33.3)	2 (28.6)	4 (30.8)
Serious	1 (16.7)	1 (14.3)	2 (15.4)
Led to discontinuation	0	1 (14.3)	1 (7.7)
With an outcome of death	0	0	0
Occurred in any patient enrolled			
Contact dermatitis	1 (16.7)	2 (28.6)	3 (23.1)
Rash	2 (33.3)	1 (14.3)	3 (23.1)
Viral upper respiratory tract infection	2 (33.3)	1 (14.3)	3 (23.1)
Increased aspartate aminotransferase	1 (16.7)	1 (14.3)	2 (15.4)
Diarrhea	2 (33.3)	0	2 (15.4)
Fatigue	0	2 (28.6)	2 (15.4)
Hyperthyroidism	1 (16.7)	1 (14.3)	2 (15.4)
Hypophosphatemia	2 (33.3)	0	2 (15.4)
Insomnia	1 (16.7)	1 (14.3)	2 (15.4)
Oropharyngeal pain	2 (33.3)	0	2 (15.4)
Pruritus	2 (33.3)	0	2 (15.4)
Abdominal pain	1 (16.7)	0	1 (7.7)
Increased alanine aminotransferase	0	1 (14.3)	1 (7.7)
Arthralgia	1 (16.7)	0	1 (7.7)
Autoimmune colitis	1 (16.7)	0	1 (7.7)
Increased blood creatinine	1 (16.7)	0	1 (7.7)
Increased blood thyroid stimulating hormone	0	1 (14.3)	1 (7.7)
Constipation	0	1 (14.3)	1 (7.7)
Contusion	1 (16.7)	0	1 (7.7)
Cough	1 (16.7)	0	1 (7.7)
Dehydration	1 (16.7)	0	1 (7.7)
Bullous dermatitis	1 (16.7)	0	1 (7.7)
Dry eye	0	1 (14.3)	1 (7.7)
Dry mouth	1 (16.7)	0	1 (7.7)
Dysgeusia	1 (16.7)	0	1 (7.7)
Eye pruritus	0	1 (14.3)	1 (7.7)
Increased gamma-glutamyltransferase	1 (16.7)	0	1 (7.7)
Hypertension	1 (16.7)	0	1 (7.7)
Hypoalbuminemia	1 (16.7)	0	1 (7.7)
Hypomagnesemia	1 (16.7)	0	1 (7.7)
Hyponatremia	0	1 (14.3)	1 (7.7)
Hypothyroidism	1 (16.7)	0	1 (7.7)
Inappropriate antidiuretic hormone secretion	0	1 (14.3)	1 (7.7)
Influenza	0	1 (14.3)	1 (7.7)
Decreased lymphocyte count	1 (16.7)	0	1 (7.7)
Lymphopenia	0	1 (14.3)	1 (7.7)
Muscular weakness	0	1 (14.3)	1 (7.7)
Neutropenia	1 (16.7)	0	1 (7.7)
Small intestinal obstruction	1 (16.7)	0	1 (7.7)
Spinal column stenosis	1 (16.7)	0	1 (7.7)
Tumor pain	1 (16.7)	0	1 (7.7)
Upper respiratory tract infection	0	1 (14.3)	1 (7.7)
Urticaria	0	1 (14.3)	1 (7.7)

*Q3W* every 3 weeks

Ten patients (76.9%) experienced at least one treatment-related AE of any grade by investigator assessment (Supplementary Table 2). The most common treatment-related AEs were rash (3/13; 23.1%), increased AST, fatigue, and hyperthyroidism (each 2/13; 15.4%). Five patients (38.5%) experienced immune-related AEs of any grade. Two patients (15.4%) experienced immune-related AEs of grade ≥ 3 of autoimmune colitis and muscle weakness (each 1/13; 7.7%); both were treated with steroids, which reduced the severity of the AEs. One additional grade ≥ 3 treatment-related AE of dehydration occurred in the same patient who had grade ≥ 3 autoimmune colitis. No dose-limiting toxicities were observed; maximum tolerated dose was not reached. No AEs led to death.

### Pharmacokinetics

**Fig. 2:**
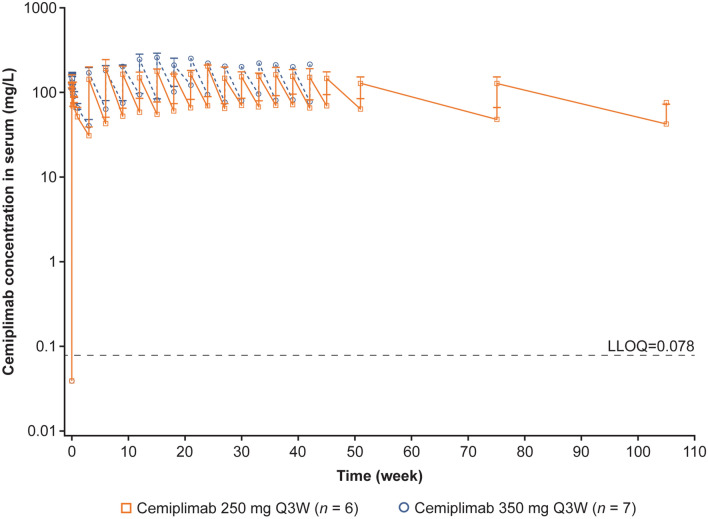
Mean (± standard deviation) of cemiplimab concentration in serum by time. *LLOQ* lower limit of quantitation, *Q3W* every 3 weeks

At steady state, mean cemiplimab concentration in serum at trough was 55.7 mg/L [standard deviation (SD) 21.8] in six Japanese patients dosed with 250 mg Q3W cemiplimab. The median body weight of these patients was 59 kg. The steady state mean cemiplimab concentrations in serum at trough was 82.6 mg/L (SD 1.6) in seven Japanese patients dosed with 350 mg Q3W cemiplimab (Fig. 2; Supplementary Table 3). The median body weight of these patients was 55 kg. Maximum cemiplimab concentrations in serum at steady state were 178 mg/L and 262 mg/L for the 250 mg Q3W and 350 mg Q3W dosing regimens, respectively.

### Immunogenicity

No ADAs were detected in serum samples collected in Part 1 of this study.

### Clinical activity

The ORR per investigator assessment was 50.0% [95% confidence interval (CI) 11.8–88.2%] in patients who received cemiplimab 250 mg Q3W, 14.3% (95% CI 0.4–57.9%) in patients who received cemiplimab 350 mg Q3W, and 30.8% (95% CI 9.1–61.4%) overall (Table 3). At the time of data cut-off, the disease control rate (DCR) was 46.2% (95% CI 19.2‒74.9%) overall. Four patients (30.8%) achieved partial response and two patients (15.4%) achieved stable disease. Of the four patients with partial response as best response, baseline diagnoses were neuroendocrine carcinoma of the lung, CSCC, leiomyosarcoma, and urachal carcinoma (each *n* = 1). Of the two patients with stable disease as best response, baseline diagnoses were squamous-cell carcinoma of unknown primary and ovarian cancer (each *n* = 1).

**Table 3:** Tumor response by investigator assessment

	Cemiplimab 250 mg Q3W (*n* = 6)	Cemiplimab 350 mg Q3W (*n* = 7)	Total (*N* = 13)
Best overall tumor response, *n* (%)			
Complete response	0	0	0
Partial response	3 (50.0)	1 (14.3)	4 (30.8)
Stable disease	1 (16.7)	1 (14.3)	2 (15.4)
Progressive disease	2 (33.3)	5 (71.4)	7 (53.8)
Objective response rate^a^ (95% confidence interval)	50.0 (11.8‒88.2)	14.3 (0.4‒57.9)	30.8 (9.1‒61.4)
Disease control rate^b^ (95% confidence interval)	66.7 (22.3‒95.7)	28.6 (3.7‒71.0)	46.2 (19.2‒74.9)

^a^Including patients with complete or partial response confirmed by repeated assessments ≥ 4 weeks apart

^b^Including patients with complete response, partial response, or stable disease; stable disease criteria were met at least once ≥ 39 days after first dose

*Q3W* every 3 weeks

**Fig. 3:**
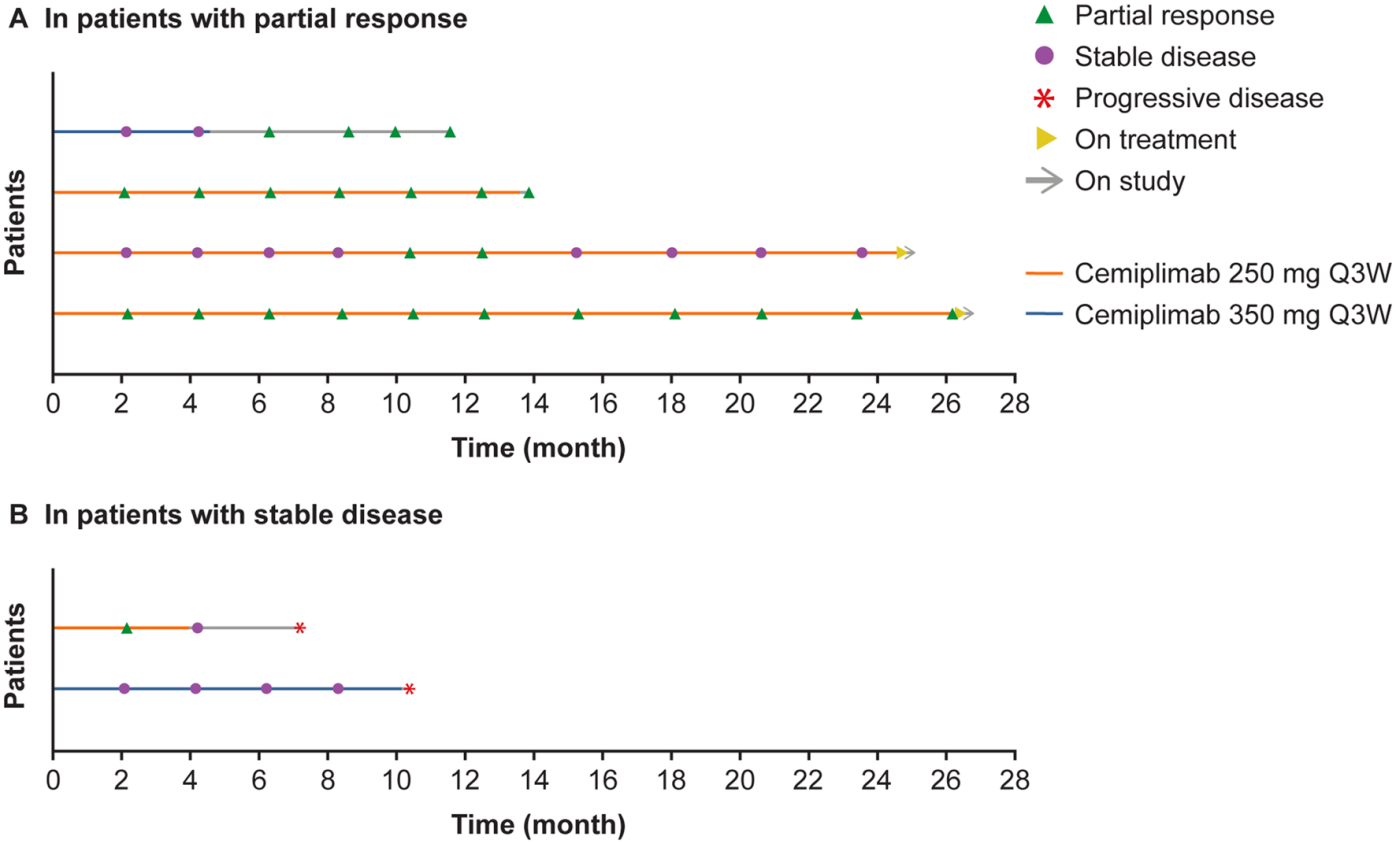
Time to and duration of response in responding patients. Plot shows time to and duration of response in **a** four patients with confirmed partial response and **b** two patients with stable disease. *Q3W* every 3 weeks

The best percent change in the sum of target lesion diameters from baseline based on investigator assessment for 11 patients who had at least one response evaluation showed tumor response to cemiplimab (Supplementary Fig. 1). Responses to cemiplimab appeared to be deep and durable (Fig. 3; Supplementary Fig. 2).

## Discussion

In Part 1 of this Phase 1 study in Japanese patients, cemiplimab showed an acceptable safety profile comparable to those reported with cemiplimab in non-Japanese patients and with other PD-1 inhibitors. Anti-tumor activity of cemiplimab in advanced malignancies was observed.

### Safety

The safety profile of cemiplimab observed in this Japanese study is consistent with that in non-Japanese studies. For example, in the cemiplimab first-in-human study, where 58 non-Japanese patients with advanced solid tumors received 1, 3, or 10 mg/kg cemiplimab Q2W (40 of these patients in the study received cemiplimab in combination with hypofractionated radiation), no dose-limiting toxicities were observed. The most common treatment-related AEs were fatigue (*n* = 14; 24.1%), arthralgias (*n* = 7; 12.1%), and nausea (*n* = 6; 10.3%). Treatment-related AEs of grade ≥ 3 were transaminase elevation (*n* = 2), anemia (*n* = 1), and anti‒Hu associated paraneoplastic encephalitis (*n* = 1) [19]. In a pivotal Phase 2 study (EMPOWER-CSCC 1), 115 non-Japanese patients with metastatic CSCC received cemiplimab monotherapy, either at 350 mg Q3W or 3 mg/kg Q2W. Of these, 113 patients (98.3%) experienced TEAEs and the most common TEAEs were fatigue (*n* = 31; 27%), diarrhea (*n* = 27; 23.5%), and nausea (*n* = 24; 20.9%). Grade ≥ 3 TEAEs occurred in 52 patients (45.2%). A total of 82 patients (71.3%) experienced treatment-related AEs of any grade and 16 patients (13.9%) experienced treatment-related AEs of grade ≥ 3 [20]. Among 21 non-Japanese patients with NSCLC who received 1, 3, or 10 mg/kg Q2W or 200 mg Q2W cemiplimab, the most common treatment-related AEs were asthenia, pneumonitis, and rash (each *n* = 3; 14.3%). Each of the following grade ≥ 3 treatment-related AEs occurred once: pneumonitis, diabetic ketoacidosis, and nephritis [5]. In 20 non-Japanese patients with recurrent or metastatic cervical cancer who received cemiplimab 3 mg/kg Q2W as monotherapy or in combination with hypofractionated radiation, the most common TEAEs of any grade were diarrhea (*n* = 4; 40.0%), fatigue, hypokalemia and pain in extremity (each *n* = 3; 30.0%) in the monotherapy cohort, and diarrhea and urinary tract infection (each *n* = 3; 30.0%) in the cemiplimab and hypofractionated radiation combination cohort [21]. No new safety signals were observed in Japanese patients.

In Japanese patient populations, the safety profile of cemiplimab is comparable to those of other PD-1 and PD-L1 inhibitors [22–24]. In a nivolumab study in 35 Japanese patients with advanced or recurrent squamous NSCLC conducted at 17 sites, 24 patients (68.6%) experienced treatment-related AEs of any grade and two (5.7%) experienced grade ≥ 3 treatment-related AEs [22]. Similarly, treatment-related AEs were reported in eight patients (80%) in a Phase 1 Japanese study of pembrolizumab in advanced solid tumors [23]. In a small dose exploration study of atezolizumab, a PD-L1 inhibitor, TEAEs occurred in all six Japanese patients with advanced solid tumors. Two patients experienced treatment-related AEs (influenza-like illness and increased alkaline phosphatase; one patient each) that led to suspension of study treatment [24].

In this Japanese study, two patients (15.4%) experienced grade ≥ 3 immune-related AEs with cemiplimab, which is consistent with data reported for non-Japanese patients. For instance, in an open-label, Phase 2 study, comprising 78 non-Japanese patients with locally advanced CSCC receiving cemiplimab 3 mg/kg Q2W, eight patients (10.3%) experienced grade ≥ 3 immune-related AEs [25]. Moreover, the incidence of grade ≥ 3 immune-related AEs with cemiplimab seems either comparable, or numerically lower than those observed with other PD-1 inhibitors in Japanese patient populations. In a retrospective analysis comprising 47 Japanese patients with metastatic renal cell carcinoma treated with nivolumab, 10 patients (21.3%) experienced grade ≥ 3 immune-related AEs [26]. Similarly, in a Phase 1 study of 42 Japanese patients with advanced melanoma treated with pembrolizumab, 13 (31.0%) patients experienced grade ≥ 3 immune-related AEs [27].

### Pharmacokinetics

The cemiplimab concentrations observed in this Japanese study were slightly higher than those observed in non-Japanese patients with advanced malignancies in previous studies, where *C*_trough_ of 58.7 mg/L and *C*_eoi_ of 166 mg/L were predicted at 350 mg Q3W at steady state by post hoc analysis [28, 29]. Considering an average body weight of 59 kg for the Japanese patients in the 250 mg Q3W cohort and 55 kg in the 350 mg Q3W cohort, compared with an average body weight of 75 kg in previous non-Japanese studies, these findings were expected since a fixed dosing regimen generated slightly higher cemiplimab concentrations in serum in patients with lower body weight [28, 29]. The impact of the higher concentrations in serum on efficacy and safety was minimal considering the wide therapeutic margin, as evidenced by relatively flat exposure–response relationships for both efficacy and safety, observed in cemiplimab and other PD-1 inhibitors [2, 30, 31]. These findings are also consistent with observations from Japanese versus non-Japanese studies of pembrolizumab and of atezolizumab [23, 24]. In addition, model-based population PK covariate analysis demonstrated that the PK of cemiplimab, nivolumab, and pembrolizumab was generally unaffected by race or geographic region [28, 29, 32, 33].

### Immunogenicity

ADA may induce infusion-related reactions or alter the PK of a PD-1 inhibitor by affecting clearance, which in turn could affect clinical activity [3, 34]. In this study, all serum samples tested negative for ADA. No infusion-related reactions were reported and response to cemiplimab treatment appeared durable. These observations agree with the minimal immunogenicity against cemiplimab observed in non-Japanese patients, in which the incidence of ADAs was 1.3% with 0.3% persistent ADA responses [14]. A review article showed that, among 10 immunogenicity analyses of nivolumab, pembrolizumab, and cemiplimab, a low incidence of ADA (0–12.7%) has been reported following single-agent treatment [3].

### Clinical activity

In this study, cemiplimab has shown clinical activity in a difficult-to-treat patient population with advanced malignancies and no alternative standard of care therapeutic option. Evidence for deep and durable tumor responses to cemiplimab was emerging. Both dosing regimens tested in this study appeared efficacious in Japanese patients. Although the ORR observed with the 250 mg Q3W dosing regimen was numerically higher than that with the 350 mg Q3W dosing regimen, there was significant overlap in the 95% CIs, indicating similar clinical activity. In addition, the slightly younger age and slightly lower ECOG performance status in the 250 mg Q3W versus 350 mg Q3W dosing groups, combined with a small patient population with diverse tumor types, might lead to high variability in clinical activity results.

The clinical activity of cemiplimab in this study is consistent with clinical activity observed in non-Japanese patient populations. In 43 non-Japanese patients with advanced solid tumors who received 1, 3, or 10 mg/kg Q2W, or 1 or 3 mg/kg Q2W cemiplimab in combination with hypofractionated radiation, partial/unconfirmed partial responses were observed in nine of 22 patients (40.9%) who received combination therapy and in two of 21 patients (9.5%) who received cemiplimab monotherapy; disease control was achieved in 27 of 43 patients (62.8%) [19]. In non-Japanese patients with metastatic CSCC treated with 350 mg Q3W or 3 mg/kg Q2W cemiplimab, the ORR and DCR were 44.3% (95% CI 33.7–53.9%) and 67.0% (95% CI 57.6–75.4%), respectively, compared to an ORR of 30.8% (95% CI 9.1‒61.4%) and a DCR of 46.2% (95% CI 19.2‒74.9%) in this study [20]. In non-Japanese patients with locally advanced CSCC who received 3 mg/kg Q2W cemiplimab, the ORR and DCR were 43.6% (95% CI 32.4–55.3%) and 79.5% (95% CI 68.8–87.8%), respectively [35]. In non-Japanese patients with metastatic or locally advanced CSCC who received cemiplimab 3 mg/kg Q2W in a Phase 1 study, the ORR and DCR were 50% (95% CI 30–70%) and 65% (95% CI 44–83%), respectively [2]. In addition, the ORR and DCR were 28.6% and 57.1%, respectively, in non-Japanese patients with NSCLC who received 1, 3, or 10 mg/kg Q2W or 200 mg Q2W cemiplimab [5]. In an analysis of 20 non-Japanese patients with recurrent or metastatic cervical cancer who received cemiplimab 3 mg/kg Q2W as monotherapy or in combination with hypofractionated radiation, ORR was 10.0% with both responders achieving ongoing responses of > 3.7 months at the time of data cut-off [21].

In Japanese patient populations, the clinical activity of cemiplimab is similar to those of other PD-1 inhibitors. Among 10 Japanese patients with advanced solid tumors in a Phase 1 study, partial responses determined by investigators according to RECIST 1.1 were observed in two patients (22.2%) treated with pembrolizumab 10 mg/kg Q2W; one patient (a 91-year-old man) had metastatic melanoma, and the other (a 53-year-old man) had NSCLC [23]. In a Phase 1 study of nivolumab in 17 Japanese patients with malignant solid tumors, complete response was observed in one patient with melanoma; partial response was observed in two patients (one with colorectal cancer; one with thyroid cancer); stable disease was observed in three patients (two with NSCLC; one with thymic cancer) [36]. In Japanese patients with previously untreated advanced melanoma who received nivolumab treatment, the ORR and DCR were 43.5% (90% CI: 28.1–60.3%) and 78.3% (90% CI: 61.6–89.0%), respectively [37].

PD-1 inhibitors, including cemiplimab, have shown remarkable clinical activity in Japanese and non-Japanese patients who progress after receiving previously established standard of care.

### Dose selection

The higher dose level, 350 mg Q3W, was selected for further analysis in Part 2 based on three considerations: similar safety profiles were observed between the two dosing regimens in Japanese patients; the significant overlap in the 95% CIs of ORRs for 250 mg Q3W and 350 Q3W indicated similar clinical activity; cemiplimab dose exploration in non-Japanese patients supported approval of the 350 mg Q3W dosing regimen for patients with metastatic or locally advanced CSCC who are not candidates for curative surgery or curative radiation in the US, Europe, Canada, Australia, Brazil, Switzerland, and Israel [13, 14, 28, 29]. This cemiplimab 350 mg Q3W dosing regimen has been selected for further development as monotherapy and in combination across disease indications.

## Study limitations

The number of patients in this Japanese study was limited. However, observations of this study in Japanese patients were generally consistent with those of previous studies in non-Japanese patients. The follow-up period of this study was relatively short as of data cut-off. Evidence of durable tumor response was emerging and will be further evaluated via longer follow-up.

## Future development

The acceptable safety profile and clinical activity observed in this Japanese study have demonstrated cemiplimab as a promising treatment option for patients with difficult-to-treat advanced malignancies and no alternative standard of care therapeutic option. Considering the observations from Japanese and global studies across multiple tumor types [2, 5, 21], cemiplimab alone or in combination is being developed for the treatment of basal cell carcinoma, lung cancer, and cervical cancer, among others.

## Electronic supplementary material

Below is the link to the electronic supplementary material.

Supplementary file1 (DOCX 450 kb)
