# Supplementary material for: Dose exploration results from Phase 1 study of cemiplimab, a human monoclonal programmed death (PD)-1 antibody, in Japanese patients with advanced malignancies
Source: Cancer Chemother Pharmacol. 2020 Nov 4;87(1):53–64. doi: 10.1007/s00280-020-04161-6 (PMC7801352; doi:10.1007/s00280-020-04161-6)
Supplement: Supplementary file 1 — Supplementary file1 (DOCX 450 kb) [file 280_2020_4161_MOESM1_ESM.docx]

# Supplementary Table 1. Exposure to cemiplimab

|  | **Cemiplimab  250 mg Q3W  (*n* = 6)** | **Cemiplimab  350 mg Q3W  (*n* = 7)** | **Total   (*N* = 13)** |
| --- | --- | --- | --- |
| Median duration of exposure, weeks (range) | 39.05 (6.0‒113.6) | 9.10 (3.0‒45.0) | 13.10 (3.0‒113.6) |
| Median number of doses administered (range) | 13.0 (2‒36) | 3.0 (1‒15) | 4.0 (1‒36) |

Q3W, every 3 weeks.

# Supplementary Table 2. Treatment-related adverse events

| ***n* (%)** | **Cemiplimab  250 mg Q3W  (*n* = 6)** | **Cemiplimab  350 mg Q3W  (*n* = 7)** | **Total   (*N* = 13)** |
| --- | --- | --- | --- |
| Any | 5 (83.3) | 5 (71.4) | 10 (76.9) |
| Occurred in any patient enrolled |  |  |  |
| Rash | 2 (33.3) | 1 (14.3) | 3 (23.1) |
| Increased aspartate aminotransferase | 1 (16.7) | 1 (14.3) | 2 (15.4) |
| Fatigue | 0 | 2 (28.6) | 2 (15.4) |
| Hyperthyroidism | 1 (16.7) | 1 (14.3) | 2 (15.4) |
| Increased alanine aminotransferase | 0 | 1 (14.3) | 1 (7.7) |
| Arthralgia | 1 (16.7) | 0 | 1 (7.7) |
| Autoimmune colitis | 1 (16.7) | 0 | 1 (7.7) |
| Increased blood creatinine | 1 (16.7) | 0 | 1 (7.7) |
| Increased blood thyroid stimulating hormone | 0 | 1 (14.3) | 1 (7.7) |
| Dehydration | 1 (16.7) | 0 | 1 (7.7) |
| Bullous dermatitis | 1 (16.7) | 0 | 1 (7.7) |
| Dry eye | 0 | 1 (14.3) | 1 (7.7) |
| Dry mouth | 1 (16.7) | 0 | 1 (7.7) |
| Dysgeusia | 1 (16.7) | 0 | 1 (7.7) |
| Eye pruritus | 0 | 1 (14.3) | 1 (7.7) |
| Increased gamma-glutamyltransferase | 1 (16.7) | 0 | 1 (7.7) |
| Hypertension | 1 (16.7) | 0 | 1 (7.7) |
| Hypomagnesemia | 1 (16.7) | 0 | 1 (7.7) |
| Hypothyroidism | 1 (16.7) | 0 | 1 (7.7) |
| Decreased lymphocyte count | 1 (16.7) | 0 | 1 (7.7) |
| Lymphopenia | 0 | 1 (14.3) | 1 (7.7) |
| Muscular weakness | 0 | 1 (14.3) | 1 (7.7) |
| Neutropenia | 1 (16.7) | 0 | 1 (7.7) |
| Pruritus | 1 (16.7) | 0 | 1 (7.7) |

Q3W, every 3 weeks.

# Supplementary Table 3. Cemiplimab concentrations in serum and pharmacokinetic parameters

| **Patient (median body weight)** | **Cemiplimab dosing regimen** | **Number of patients after first dose** | **Mean (standard deviation)** | | | | | | | |
| --- | --- | --- | --- | --- | --- | --- | --- | --- | --- | --- |
|  |  |  |  |  | **After first dose** | | **After 3 doses** | | **At steady state** | |
|  |  |  | **AUC_tau_**  **(mg/L×d)** | **T_1/2_**  **(day)** | **C_trough_**  **(mg/L)** | **C_eoi_**  **(mg/L)** | **C_trough_**  **(mg/L)** | **C_eoi_**  **(mg/L)** | **C_trough_**  **(mg/L)** | **C_eoi_**  **(mg/L)** |
| Japanese  (59 kg) | 250 mg Q3W | 6 | 1089 (86.7) | 23.9 (26.1) | 31.3 (7.10) | 110.8 (23.1) | 52.5 (12.3) | 194.2 (50.3) | 55.7 (21.8) | 178 (13.9) |
| Japanese  (55 kg) | 350 mg Q3W | 7 | 1293 (198.3) | 16.44 (3.38) | 41.0 (6.9) | 145.1 (24.8) | 75.2 (4.3) | 182.8 (28.2) | 82.6 (1.6)* | 262 (32.5)* |

*Data from two patients.

AUC_tau_, area under the curve at end of dosing period; C_eoi_, maximum concentration; C_trough_, minimum concentration; Q3W, every 3 weeks; T_1/2_, half-life.

# Supplementary Fig. 1. Tumor response to cemiplimab by investigator assessment


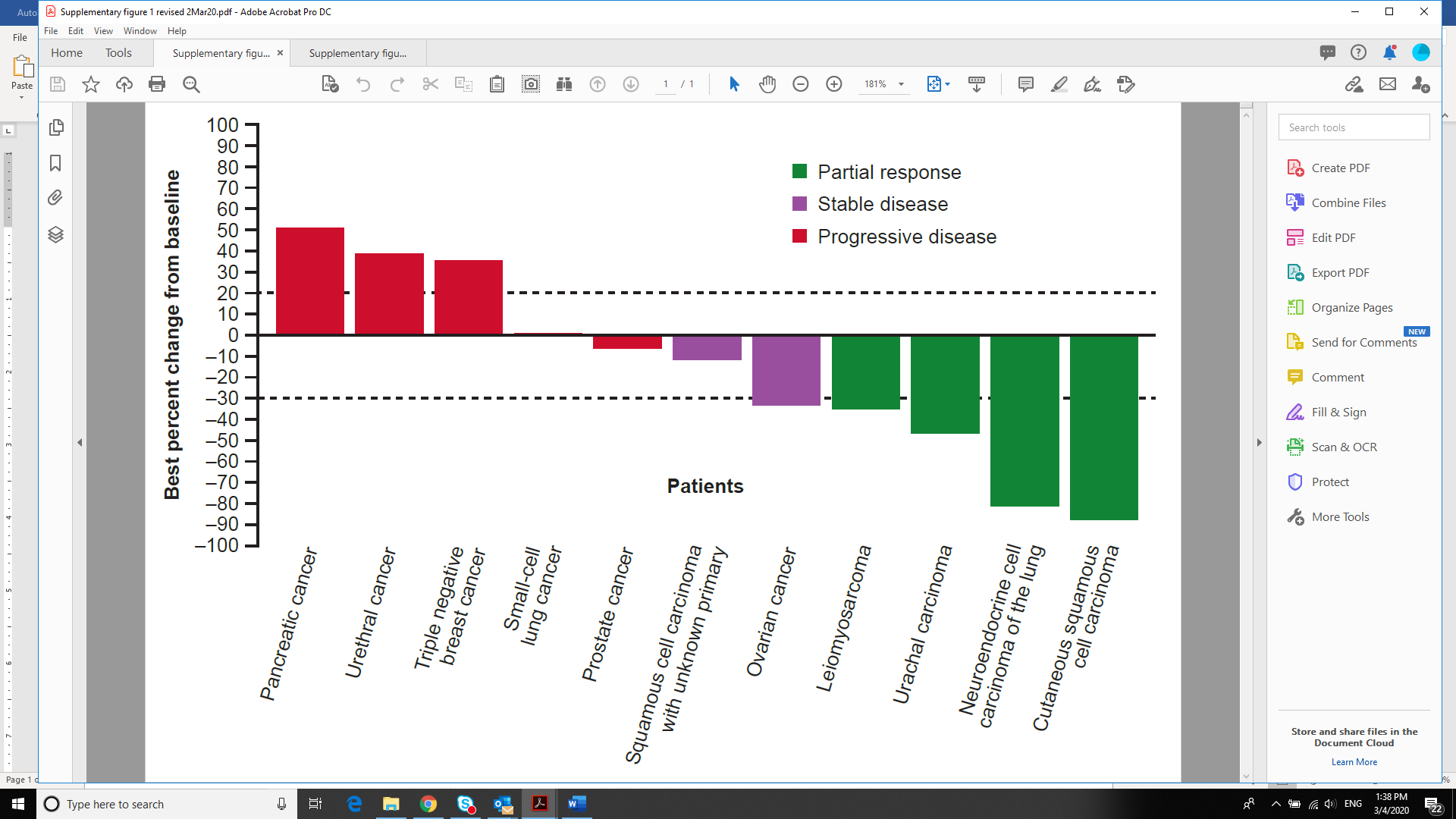


Plot shows the best percentage change in the sum of target lesion diameters from baseline for 11 patients who had at least one response evaluation. Two patients with non-target lesions were not included; both patients had best overall response of progressive disease. Lesion measurements after progression were excluded. Horizontal lines indicate criteria for partial response (≥30% decrease in the sum of target lesion diameters) and progressive disease (≥20% increase in the target lesion diameters), respectively.

# Supplementary Fig. 2. Change in target lesion over time by investigator assessment


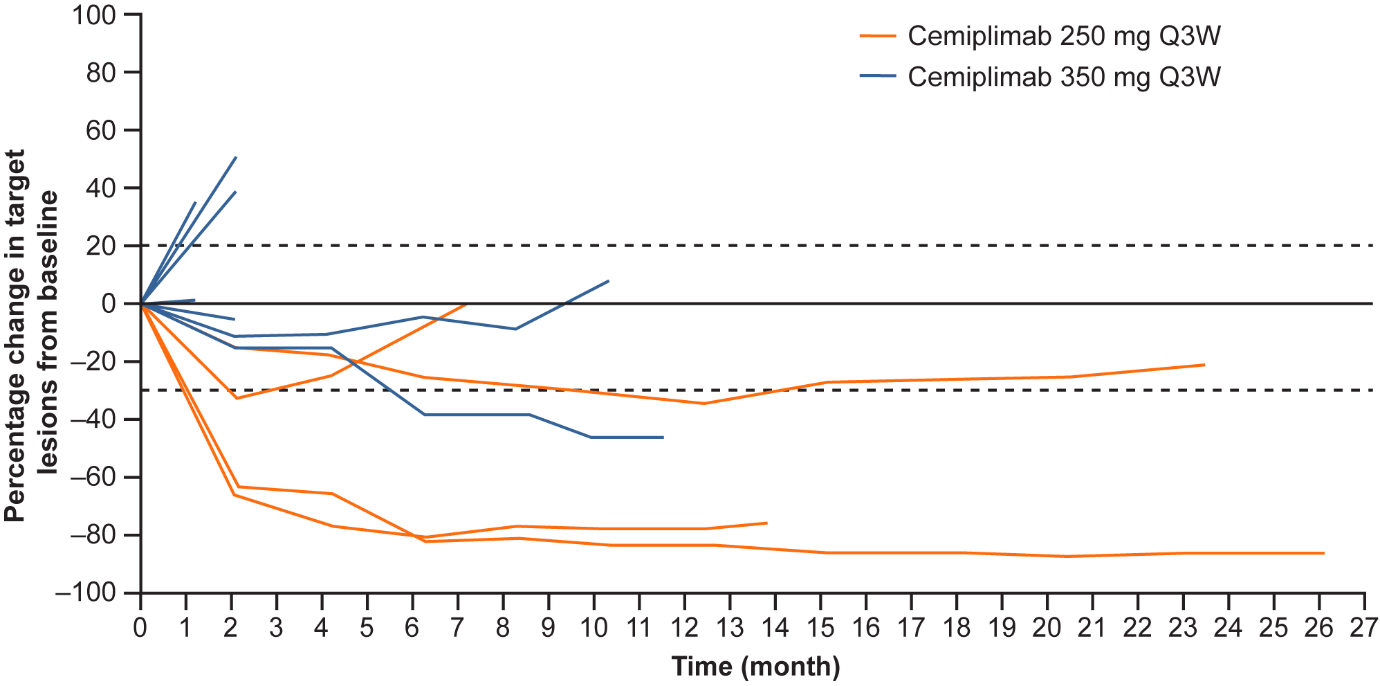


Plot shows the percent change in target lesions from baseline over time in 11 patients who had at least one response evaluation. Two patients with nontarget lesions were not included; both patients had best overall response of progressive disease. The horizontal dashed lines indicate criteria for partial response (≥30% decrease in the sum of target lesion diameters) and progressive disease (­20% increase in the target lesion diameters).

Q3W, every 3 weeks.
